# Fandom Biases Retrospective Judgments Not Perception

**DOI:** 10.1038/srep43083

**Published:** 2017-02-24

**Authors:** Markus Huff, Frank Papenmeier, Annika E. Maurer, Tino G. K. Meitz, Bärbel Garsoffky, Stephan Schwan

**Affiliations:** 1 Department of Psychology, Eberhard Karls Universität, Tübingen, Germany; 2 Leibniz Science Campus, Tübingen, Germany; 3 Leibniz-Institut für Wissensmedien, Tübingen, Germany

## Abstract

Attitudes and motivations have been shown to affect the processing of visual input, indicating that observers may see a given situation each literally in a different way. Yet, in real-life, processing information in an unbiased manner is considered to be of high adaptive value. Attitudinal and motivational effects were found for attention, characterization, categorization, and memory. On the other hand, for dynamic real-life events, visual processing has been found to be highly synchronous among viewers. Thus, while in a seminal study fandom as a particularly strong case of attitudes did bias judgments of a sports event, it left the question open whether attitudes do bias prior processing stages. Here, we investigated influences of fandom during the live TV broadcasting of the 2013 UEFA-Champions-League Final regarding attention, event segmentation, immediate and delayed cued recall, as well as affect, memory confidence, and retrospective judgments. Even though we replicated biased retrospective judgments, we found that eye-movements, event segmentation, and cued recall were largely similar across both groups of fans. Our findings demonstrate that, while highly involving sports events are interpreted in a fan dependent way, at initial stages they are processed in an unbiased manner.

In a seminal study, it was shown that fandom influenced retrospective judgments about an American football match^1^. Although this study just reported retrospective judgments, this effect is often interpreted as evidence for selective perception. In fact, attitudes and motivations have been shown to affect the processing of visual input, indicating that observers may see a given situation each literally in a different way^2^,^3^. Yet, processing real-life information in an unbiased manner is considered to be of high adaptive value^4^,^5^. Although attitudinal and motivational effects were found for attention^6^, characterization^7^, categorization^8^, and memory^9^, visual processing of dynamic real-life events has been found to be highly synchronous among viewers^10^,^11^,^12^.

We studied attention, perception, memory, and retrospective judgments of real football fans while they watched the live TV broadcasting of the 2013 UEFA Champions League Final. This enabled to test the hypothesis that fandom influences not just retrospective judgements but also prior processing stages using the same stimulus material within a single study.

The 2013 UEFA Champions League Final, the focus of this study, was particularly emotionally charged because the opponents in this international final were the two archrival German football (soccer) teams Borussia Dortmund (BVB) and FC Bayern München (FCB). BVB dominated the first 30 minutes of the match. FCB later took the initiative and scored 1:0 in the 60th minute. Shortly after, in the 69th minute, BVB scored a penalty kick (1:1). FCB scored the winning goal (1:2) with one minute left within normal time. On average, 125 million people worldwide watched the match on TV^13^. Thus, we had the unique opportunity to study influences of fandom on perception^1^ of a highly involving dynamic event, with both fan groups having opposing attitudes in favor of their team. Importantly, both fan groups were able to watch the very same TV broadcast. One hundred thirty-two fans applied for participation in our study. Fifty-eight of these fans (33 supporting BVB and 25 supporting FCB) with the highest scores on a questionnaire asking for interest in football and fandom were invited to take part. Each of them watched the live TV broadcast of the game in separate cubicles on a separate screen, thus ensuring equal viewing conditions. Covering different processing steps of event perception, we measured affective states before, during (i.e. in the half-time break) and after the game, eye-movements and segmentation behavior during the game, performance and confidence in a cued recall test immediately and ten to twelve days after the game, as well as summative retrospective judgments ten to twelve days after the game.

We expected fandom to influence participants’ affective states accordingly. To check for differences in affective states between fan groups, we used the positive and negative affect schedule (PANAS), a standard questionnaire in affective research^14^,^15^. This questionnaire assesses participants’ attentive states such as “attentiveness”, “fear”, “hostility”, “negative affect”, “positive affect”, and “self-assurance” (Note: due to technical problems, 51 participants entered this analysis). During the match, both fan groups showed high values of attentiveness and positive affect, indicating a high degree of involvement, reflecting the balanced course of the game. Immediately after the match, FCB fans showed higher values on the subscales “attentiveness”, “positive affect”, and “self-assurance”. In contrast, BVB fans showed higher values on the subscale “negative affect” (all *p*s < 0.006, Fig. 1a and Table 1); that is to say, affective states varied according to the outcome of the game (FCB won the game with the deciding goal in the last minute), providing further evidence that our participants were highly involved and biased according to their fandom. In the delayed test (ten to twelve days after the game), we no longer observed any differences between fan groups.

We expected fans’ retrospective judgments of the game to be biased towards their own team^1^,^16^. In the delayed test, we asked participants to indicate how much each team contributed to the game with regard to ball possession, tackles won, and scoring chances. Corroborating our hypothesis and replicating prior work^1^, FCB’s contribution to the game was rated higher by FCB fans as compared to BVB fans, *t*(49) = 2.89, *p* = 0.006, *d* = 0.83 (Fig. 1b).

Turning to the prior processing stages, we first analyzed eye-movements. Eye-movements are not only influenced by stimulus’ characteristics^12^,^17^ but also by viewers’ tasks, goals, and attitudes^18^, with dynamic scenes being less influenced by the latter^19^. If fandom biases processes as early as eye gaze differences, we should find differing gaze patterns between fan groups. We recorded participants’ eye-movements during the game (due to technical problems *N* = 15 of originally *N = *21 and only the first half-time entered the final analysis). We used the normalized scanpath saliency (NSS)^20^ to measure the coherence of each participant’s eye-movements within and across the two fan groups (Fig. 1c). The more similar (coherent) the gaze of a participant and group is, the higher the resulting values are. To analyze the data statistically, we subtracted the NSS value that was based on the opposing fan group as the reference group from the NSS value that was based on the same fan group as the reference group for each participant and time point. Thereafter, we aggregated these difference scores across time, giving us a mean difference score for each participant. If fan group membership reliably biases gaze behavior, this difference score should be above zero. However, a t-test revealed that those difference scores (*M* = −0.03, *SD* = 0.27) did not significantly differ from zero, *t*(14) = −0.46, *p* = 0.655, *d* = 0.12. This is further supported by the Bayes factor evidence for the null hypothesis, which amounted to 5.15 (conventionally classified as “substantial”)^21^ in a one-tailed Bayesian t-test comparing the difference scores against 0. Thus, we could show that gaze behavior was not biased by fandom.

Event segmentation is a means to measure perceptual and cognitive processing of dynamic events by asking observers to press a button to indicate when in their opinion one meaningful event ends and another meaningful event begins. There is compelling agreement among participants about the temporal location of such event boundaries^22^,^23^,^24^. Event segmentation behavior is based on both bottom-up^25^,^26^ and top-down^27^ processing of the features of the behavioral event stream. Top-down influences – such as observers’ task goals – were only observed in case participants were instructed to segment the dynamic event into natural units or received no specific instruction^27^,^28^,^29^. Directing participants’ attention towards specific aspects of the event – as it is the case when instructing participants to segment the depicted action into fine and/or coarse events–presumably prevents influences of top-down processes on segmentation^30^. We thus employed a natural event segmentation task asking participants to segment the game into events that seemed natural and meaningful to them. During the game, thirty-seven participants performed an event segmentation task^31^,^32^. Due to technical problems, we lost segmentation data of 3 participants, thus, data of 34 participants entered the segmentation analysis. For each participant and each time point of a button press, we selected the segmentation magnitude score of their own corresponding fan group (BVB for BVB fans and FCB for FCB fans; we excluded the respective participant from the segmentation magnitude score to avoid the problem of autocorrelation) and the segmentation magnitude score of the corresponding other fan group (FCB for BVB fans and BVB for FCB fans). This resulted in two values for each participant and button press, namely the “segmentation agreement with one’s own fan group” and the “segmentation agreement with the opposing fan group”. We then calculated the difference between these two scores for each button press. We aggregated these difference scores across the button presses, giving us a mean difference score for each participant. This allowed us to compare consistency in event segmentation behavior across fan groups. If fandom biased event segmentation behavior, we expected to find a difference score significantly larger than zero indicating that participants’ segmentation behavior was more consistent with the own fan group than the other fan group. However, if event segmentation was a bottom-up process, that is, unbiased by fandom, we expected to find a difference score that does not significantly differ from zero, which is what we found, *t*(33) =−1.49, *p* = 0.144, *d* = 0.26. This conclusion is supported by the Bayes factor evidence for the null hypothesis, which amounted to 12.50 in a one-tailed Bayesian t-test comparing the difference scores against 0. Thus, we conclude that segmentation behavior was also not biased by fandom.

Human memory is sensitive to biases like stereotypes^9^, thus, if fandom biased memory in a similar way, we expected memory performance to be dependent on fandom. Yet, we found no influence of fandom on performance in a cued recall task testing participants’ memory immediately and ten to twelve days after the game. The test comprised items from the match, depicting a central event (e.g., a goal shot or a free kick). Participants watched the 7-sec video clips and indicated whether ball possession did change immediately after or not. Each item’s distance to the next event boundary was calculated before the analysis. This was important because event perception literature consistently reports that memory for situations at event boundaries is higher than memory at non-event boundaries^33^. Performance declined with increasing distance to an event boundary (Fig. 2), *F*(2,108) = 19.83, *p* < 0.001, η_p_^2^ = 0.27, thus replicating basic effects of event perception research^33^. The significant interaction of “distance” and “team in ball possession”, *F*(2,108) = 20.01, *p* < 0.001, η_p_^2^ = 0.27, indicated that the distance effect was more pronounced for BVB items than for FCB items. This interaction was more pronounced in the delayed test as indicated by the significant three-way interaction of “distance”, “team in ball possession”, and “test time”, *F*(2,108) = 4.85, *p* = 0.010, η_p_^2^ = 0.08. However and most important, performance was unaffected by fandom as indicated by the non-significant “team in ball possession” x “fan” interaction, *F*(1,54) = 0.13, *p* = 0.725, η_p_^2^ < 0.01 (see Fig. 1d). Accordingly, fandom did not bias cued recall test performance, which is further supported by a Bayes factor ANOVA including “team in ball possession” and “fan” as factors and default prior scales. This analysis revealed that the main effects model was preferred to the interaction model by a Bayes factor of 8.16.

It is still a matter of debate whether performance and confidence in a memory test are interrelated. Evidence from research on flashbulb memories suggest that these are separate processes^34^. Therefore, we also asked participants to give a confidence rating after each response in the cued recall test. In line with memory performance, confidence declined with increasing distance to the event boundary, *F*(2,108) = 70.91, *p* < 0.001, η_p_^2^ = 0.57. This was less pronounced in the delayed test as indicated by the interaction of “distance” and “test time”, *F*(2,108) = 6.58, *p* = 0.002, η_p_^2^ = 0.11 (Fig. 2). In contrast to memory performance, we found that participants’ subjective confidence ratings were biased by fandom immediately after the match and in the delayed test. Each fan group was more confident in responding to items depicting their own team in ball possession as compared to items depicting the opposing team in ball possession as indicated by a significant fan x team interaction, *F*(1,54) = 9.52, *p* = 0.003, η_p_^2^ = 0.15 (Fig. 1d).

Unpredictable and rapidly unfolding dynamic scenes–there was a total of 839 passes in this game–require viewers to pay constant attention to the most relevant parts; otherwise, they will miss important aspects (such as a pass interception or a foul). Our results show that fandom biases subjective judgments (such as recollection about the relative contribution of the teams and confidence in the cued recall test) but not basic perceptual and cognitive processes (such as eye-movements, event segmentation, and performance in the recall test).

Based on empirical findings^27^,^28^ and theoretical considerations^22^, we hypothesized that fandom biases basic perceptual and cognitive processes. This first test of this hypothesis has clearly shown that judgments but not online processing is biased by fandom^1^,^2^,^6^. This is in line with recent research demonstrating that reported top-down influences are sometimes falsely attributed to perception instead of judgments^5^. Because fandom strongly influenced participants’ affective states and–at the same time–there were no effects on participants’ gaze and event segmentation behavior, the present findings support the claim that the physical structure of dynamic events might be sufficient to guide observers’ event perception^35^.

In the present study, we employed a natural event segmentation task that has proven to be sensitive to top-down influences in the past^27^,^28^. Event segmentation requires participants to make explicit decisions about when to press the button. Recently, it was shown that gaze behavior is modulated as a function of the event structure^36^ and is further influenced by participants’ attitudes^18^. Because we did not find a difference in the eye tracking data either, we conclude that event perception (as measured in the present study) was not biased by fandom. More research on the role of top-down processes is necessary to specify the effect of fandom on further measures associated with online processes when watching highly dynamic events. In this light, we expect our experimental approach to be a starting point for more sophisticated theoretical models of the influence of attitudes on perceptual and cognitive processes. For example, distinguishing *perceptual judgments* (e.g., confidence) from the *perception of events* (e.g., event segmentation or cued recall performance) might help to resolve contradicting evidence on the relation between attitudes and perception^1^,^2^,^5^,^6^,^22^,^37^. Based on our results, we anticipate that attitudes bias the former but not the latter.

## Methods

### Ethical approval and informed consent

The research was conducted in accordance with APA (American Psychological Association) standards for ethical treatment of participants and with approval of the institutional review board of the Leibniz-Institut für Wissensmedien (IWM), Tübingen. All participants provided written informed consent before participating in this study.

### Participants

Participants were invited to apply for participation via a newspaper announcement and an e-mail sent to all students and employees of the University of Tübingen. Applicants were asked to fill in an internet-based questionnaire with football related questions (e.g., which team they support, how often they watch football on TV). Based on these answers, we selected 58 fans out of 132 applicants; 33 supported BVB and 25 supported FCB. Their first language was German. All participants gave informed consent and received a monetary compensation of 40 Euros.

### Apparatus, stimuli, and procedure

The experiment was programmed using PsychoPy^38^. Stimulus material was the live TV broadcast of the 2013 *UEFA Champions League Final*. Each participant sat in a separate cubicle equipped with a notebook computer. A dedicated server streamed the TV signal to each notebook, thus ensuring that all notebooks received the signal at the same time. In the “segmentation cubicles”, one key of the keyboard was marked to indicate which button to press when they perceived a meaningful boundary between two events. The “eye-tracking cubicles” were equipped with video-based eye-tracking devices, 15 × SMI RED-m, 5 × SMI RED, 1 × SMI RED-250, measuring participants gaze at 120 Hz, 60 Hz, and 250 Hz, respectively. For the purpose of the analysis, all data was converted to 60 Hz.

Participants’ affective states were measured with the German version of the Positive Affect Negative Affect Schedule (PANAS) using the subscales “attentiveness”, “fear”, “hostility”, “negative affect”, “positive affect”, and “self-assurance”^14^,^15^. Internal consistency was high, Cronbach’s α = 0.75, 95% CI [0.63, 0.86]. The PANAS scores for “attentiveness”, “hostility”, “negative affects”, “positive affects”, and “self-assurance” showed diverging affective states of the two fan groups in the course of the game. We analyzed the data with separate mixed factor ANOVAs with fan group (BVB, FCB) as between-subjects factor and time (0: before, 1: half-time break, 2: immediately after the game, 3: ten days after the game) as within-subjects factor for each sub-scale.

### Eye-tracking

**Figure 1:**
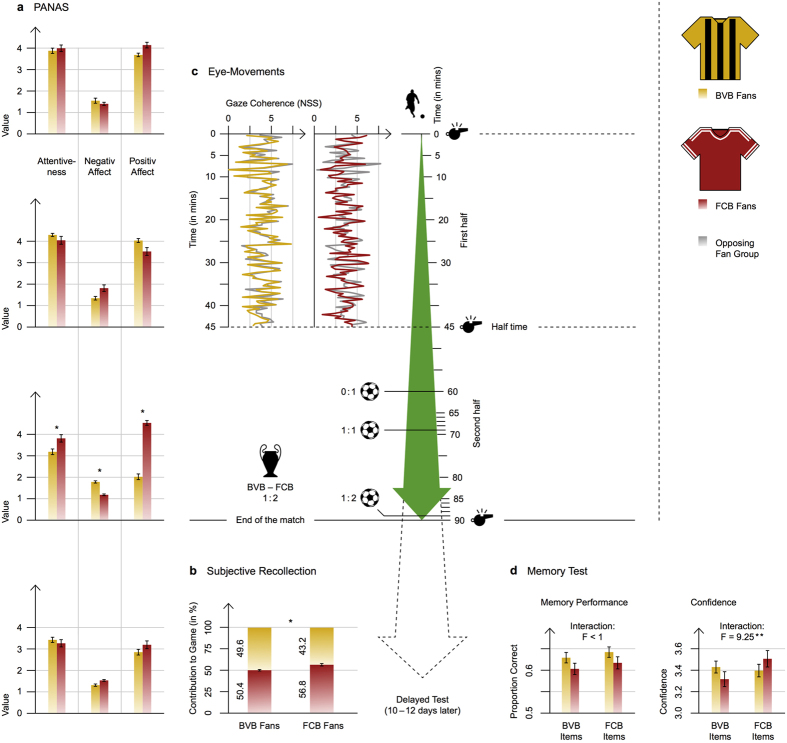
Main results of the reported study. (**a**) PANAS scores before the game, in the half-time break, as well as in the immediate and delayed test. (**b**) Subjective recollection of the proportion that each team contributed to the game as a function of fandom. (**c**) Gaze coherence (NSS) across the first half-time for BVB fans (top)/FCB fans (bottom) calculated based on either the same fan group or opposing fan group as reference group. The figure shows the lowess smoother lines (smoother span: 0.001) of the raw NSS values in 40 ms steps. The overlap of both lines indicates that participants’ gaze was equally coherent with the same fan group and opposing fan group, thus indicating that fandom did not bias gaze behavior. (**d**) Participants’ performance and confidence ratings in the memory test. All error bars represent the standard error of the mean (*SEM*).

The gaze of 21 participants was recorded. We used a 9-point calibration and placed participants at an unrestricted viewing distance of 65 cm from the screen. We removed all participants with a calibration accuracy larger than 0.8 deg of visual angle (first half-time: 5 participants; second half-time: 9 participants). In addition, the eye-tracking data of one participant was lost due to technical problems. Because this left us with a too small sample for the second half-time (4 BVB fans, 7 FCB fans), we restricted our analysis to the first half-time (6 BVB fans, 9 FCB fans). We collected eye-tracking data in order to investigate whether the two fan groups had actually watched the same game or different games. More formally, we were interested whether fan group membership causes distinct gaze patterns. Therefore, we analyzed whether the gaze pattern of participants can be better predicted based on participants sharing the same fan group membership than based on participants that are fans of the opposing team. In order to answer this question, we used the normalized scanpath saliency (NSS) that has recently been adapted for measuring gaze coherence of multiple participants in dynamic scenes^20^. In short, with NSS one calculates gaze coherence of a group by running the following calculation for each participant and time point: 1) create a fixation map in space and time from the gaze positions of the other group members, 2) normalize the fixation map, and 3) insert the gaze position of the respective participant into the normalized map. The more similar (coherent) the gaze of the participant and group is, the higher the resulting values are. The individual NSS values can then be combined to a mean NSS value describing the coherence of the group at the specific point in time (see Fig. 1c). Please note that we based the normalization of the fixation map on the values of the fixation map at each respective point in time instead of taking all of the values from a spatiotemporal window around this point in time into account^20^. This was done to prevent distortions caused by the latter method when abrupt changes in gaze coherence occur^39^. We adapted the NSS method to our research question by not only calculating the fixation map based on the group members sharing the same fan group membership as a reference group, but also by using a random sample of n-1 members of the other fan group as a reference group. We used n-1 random participants of the other fan group to account for the fact that the usual NSS calculations also use n-1 members of the same fan group because the respective participant is removed from the group when calculating the fixation map. Doing so, we calculated NSS values that indicate the similarity of participants’ gaze with either the same fan group or opposing fan group. If fan group membership causes distinct gaze patterns, we should observe higher NSS values when the reference group consists of the same fan group than when it consists of the opposing fan group.

We applied this analysis to the gaze data of the first half-time in 40 ms steps. In order to ensure appropriate data quality, we removed those NSS values where the fixation map was calculated based on less than four participants or the group’s mean NSS value was based on less than four values due to missing gaze data (2.3% of the data). Furthermore, the participant level analysis was performed across all combinations of participant and time point, fulfilling the following criterion: that the availability of a valid gaze position for the participant and both fixation maps (each reference group respectively) were based on at least four reference participants during the calculation of the participant-level NSS value at the given time point. Thus, this analysis included 90.4% of the data.

**Figure 3:**
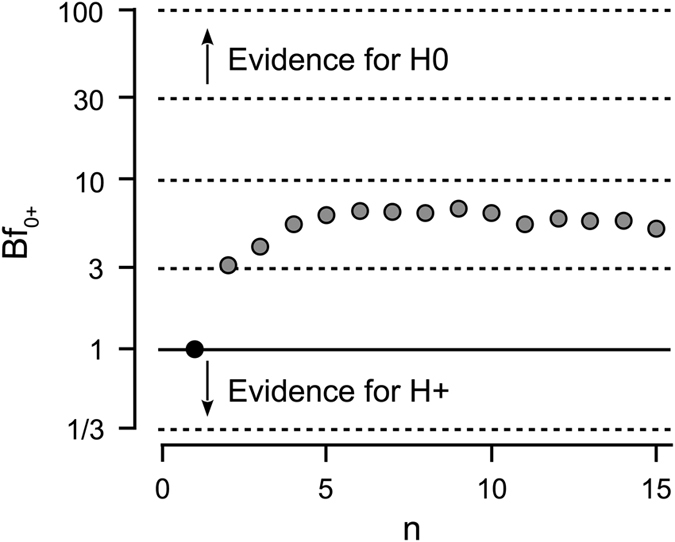
Sequential analysis showing the progression of the Bayes factor in favor of the null hypothesis as new participants enter the analysis.

Because of the sample size of only 15 participants, who finally entered the analysis, we calculated a sequential analysis using the JASP software package^40^. The sequential analysis is a means to illustrate how the Bayes factor develops with each additional participant entering analysis. Thus, this analysis explores the robustness of an effect^41^. As can be seen on Fig. 3, the evidence in favor of the null hypothesis (Bf = 5.15) proves stable starting from six participants in the analysis.

### Segmentation

Thirty-seven participants performed an event segmentation task^32^: They watched the football match and pressed a button to indicate when, in their opinion, one meaningful unit ended and another one began. The instruction stated the following:

*“(…) In this part of the experiment, we will ask you to perform the following task while watching the football match. Please segment the game into units that seem natural and meaningful to you. There is no right or wrong manner of doing this: We are just interested in the way you perform this task. To mark the boundaries between two units, please press the yellow button. Please make sure you press the button as close as possible to the end of a unit. Do not press the button in the middle of a unit. Please remember that there is no right or wrong way to mark these units; just make sure to press the button when – in your opinion – a meaningful unit ends and another one begins. (…)”*.

Due to technical problems, we lost segmentation data of 3 participants, thus, data of 34 participants entered the segmentation analysis. To describe segmentation behavior across all participants who segmented the football game into meaningful events, Gaussian distribution functions were estimated around each response (i.e. key press). The standard deviation was set to 1000 ms, that was estimated and validated using the data of a recent experiment with animated football scenes^23^. We summed up the resulting Gaussian distributions for each fan group and half-time separately and normalized the resulting time series by the number of participants that were included.

### Performance and confidence in the memory test

The cued recall test consisted of a total of 52 filmic items depicting central events of the match. These events were determined during the match using the following rationale: all shots on targets, all corner kicks, and other (highly salient) critical situations (e.g., free kicks following fouls). The video clips were seven seconds in length and were masked at the moment the ball was up in the air. Participants were required to indicate with a button press whether ball possession changed or not and how confident they were about this (5-point scale: 1: “very unconfident” … 5: “very confident”).

We excluded two participants who did not complete the delayed cued recall test. Thus, the memory test analysis contained data from 56 participants (32 BVB and 24 FCB fans). We determined significant event boundaries and the temporal distance of the memory items to the next event boundary. We used bootstrapping methods to determine the respective 95%-criterion for the definition of significant event boundaries based on the segmentation data as described above^30^. These event boundaries are important to describe the items of the memory test because memory for situations at event boundaries is generally higher than memory at non-event boundaries^26^. Based on these data, we omitted five memory items with a distance to the next event boundary being larger than 200 seconds (box plot criterion). The remaining items were divided into the tertiles [0, 5.1], (5.1, 42.0], and (42.0, 189] seconds distance to the next event boundary.

#### Performance

We analyzed event memory in terms of proportion correct and aggregated the data on the participant level. We submitted the data to a mixed-factor ANOVA, including the within-subject factors “team in ball possession” (BVB, FCB), “distance of test item to the next event boundary” (tertiles), and “test time” (immediate, delayed), as well as the between-subjects factor “fan” (BVB, FCB). Apart from the reported main effects and interaction, we observed no other significant main effects and interactions; that is, fandom did not bias memory performance. Taken together, we conclude that memory for event boundary items is higher than memory for non-event boundary items, thus replicating previous research. However, items depicting one’s own team in ball possession were not remembered more precisely. Thus, memory was not biased towards one’s own team.

#### Confidence

We repeated this analysis using “confidence” (1: “very unconfident”, up to 5: “very confident”) as dependent variable (Fig. 1d). In addition to the reported effects, no other effect reached the level of significance, *F*s ≤ 3.23, *p*s* ≥* 0.063, η_p_^2^s ≤ 0.06. In summary, the results of the confidence ratings complement those of the proportion correct analysis; confidence declines with increasing distance to the next event boundary. Most important, fan group membership interacted with the content of the memory items. Confidence was higher for those items depicting the supported team in ball possession.

### Procedure

Participants arrived about 60 minutes before the start of the match, gave informed consent, and were then guided to their respective cubicle. We assigned participants not wearing glasses or lenses to the eye-tracking condition. All participants then received information about their own football team (e.g., latest successes, club emblem) and filled out the PANAS questionnaire for the first time. During the half-time break, participants filled out the PANAS questionnaire again and received light refreshments. The participants in the eye-tracking condition were re-calibrated right before the start of the second half. After the match had ended, participants filled out the PANAS questionnaire for the third time and completed the cued recall test. Participants ultimately returned to the lab ten to twelve days after the match, filled out the PANAS questionnaire for the last time and completed the cued recall test a second time.

## Additional Information

**How to cite this article:** Huff, M. *et al*. Fandom Biases Retrospective Judgments Not Perception. *Sci. Rep.* **7**, 43083; doi: 10.1038/srep43083 (2017).

**Publisher's note:** Springer Nature remains neutral with regard to jurisdictional claims in published maps and institutional affiliations.

## Figures and Tables

**Figure 2:**
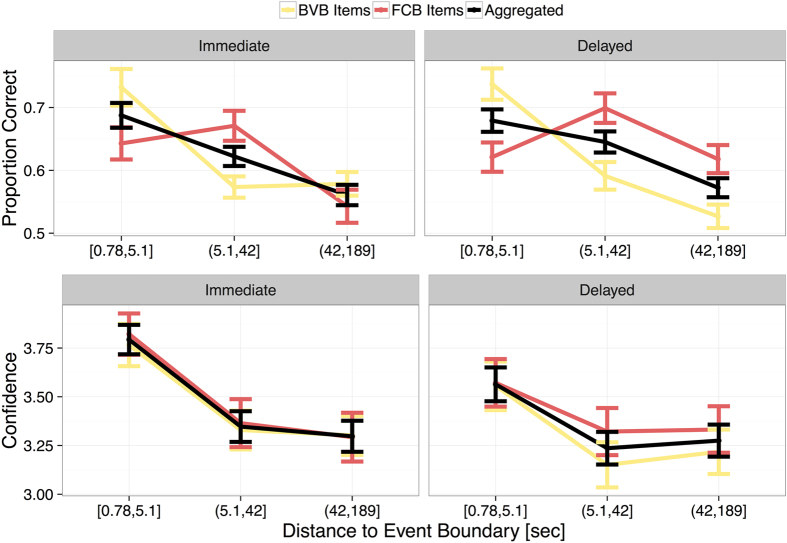
Performance in the immediate and delayed cued recall test (upper row) and confidence rating results (lower row) as a function of temporal distance to the event boundary and test time and team. The colored lines represent the items depicting either BVB or FCB in action; the black lines represent the aggregated values. Error bars indicate the *SEM*.

**Table 1:** Results of the PANAS subscales.

	Fan	Time	Time x Fan
Attentiveness	*F* < 1	*F*(3,147) = 17.97, *p* < 0.001, η_p_^2^ = 0.27	*F*(3,147) = 4.15, *p* = 0.007, η_p_^2^ = 0.08
Fear	*F* < 1	*F*(3,147) = 23.91, *p* < 0.001, η_p_^2^ = 0.33	*F* < 1
Hostility	*F*(1,49) = 7.00, *p* = 0.011, η_p_^2^ = 0.12	*F*(3,147) = 4.76, *p* = 0.003, η_p_^2^ = 0.09	*F*(3,147) = 5.57, *p* = 0.001, η_p_^2^ = 0.10
Negative Affect	*F*(1,49) = 2.90, *p* = 0.095, η_p_^2^ = 0.06	*F*(3,147) = 11.68, *p* < 0.001, η_p_^2^ = 0.19	*F*(3,147) = 6.04, *p* < 0.001, η_p_^2^ = 0.11
Positive Affect	*F*(1,49) = 26.30, *p* < 0.001, η_p_^2^ = 0.35	*F*(3,147) = 33.50, *p* < 0.001, η_p_^2^ = 0.41	*F*(3,147) = 54.10, *p* < 0.001, η_p_^2^ = 0.52
Self-Assurance	*F*(1,49) = 1.93, *p* = 0.170, η_p_^2^ = 0.04	*F* < 1	*F*(3,147) = 17.56, *p* < 0.001, η_p_^2^ = 0.26
